# Muscle-strengthening Exercise Epidemiology: a New Frontier in Chronic Disease Prevention

**DOI:** 10.1186/s40798-020-00271-w

**Published:** 2020-08-26

**Authors:** Jason A. Bennie, Jane Shakespear-Druery, Katrien De Cocker

**Affiliations:** Physically Active Lifestyles Research Group (USQ PALs), Centre for Health Research, Institute for Resilient Regions, University of Southern Queensland, Education City, 37 Sinnathamby Boulevard, Springfield Central, Queensland 4300 Australia

**Keywords:** Strength training, Exercise, Public health, Health surveillance, Physical activity

## Abstract

This current opinion provides an overview of the emerging discipline of muscle-strengthening exercise epidemiology. First, we define muscle-strengthening exercise, and discuss its recent addition into the global physical activity guidelines, which were historically mainly focused on aerobic physical activity (walking, running, cycling etc.). Second, we provide an overview of the current clinical and epidemiological evidence on the associations between muscle-strengthening exercise and health, showing a reduced mortality risk, and beneficial cardiometabolic, musculoskeletal, functional and mental health-related outcomes. Third, we describe the latest epidemiological research on the assessment, prevalence, trends and correlates of muscle-strengthening exercise. An overview of recent population estimates suggests that the proportion of adults meeting the current muscle-strengthening exercise guideline (10-30%; ≥ 2 sessions/week) is far lower than adults reporting meeting the aerobic exercise guideline (~ 50%; ≥ 150 min/week). Fourth, we discuss the complexity of muscle-strengthening exercise promotion, highlighting the need for concurrent, coordinated, and multiple-level strategies to increase population-level uptake/adherence of this exercise modality. Last, we explore key research gaps and strategies that will advance the field of muscle-strengthening exercise epidemiology. Our objective is to provide a case for increased emphasis on the role of muscle-strengthening exercise for chronic disease prevention, and most importantly, stimulate more research in this currently understudied area of physical activity epidemiology.

## Key Points

- Whilst clinical and epidemiological evidence links muscle-strengthening exercise to optimal health and well-being, over 80% of adults do not report meeting the muscle-strengthening exercise guidelines (≥ 2 times/week).
- Compared to aerobic physical activity/exercise, muscle-strengthening exercise has been generally overlooked in public health approaches for chronic disease prevention.
- Future research in muscle-strengthening exercise epidemiology should focus on standardising assessment instruments and assessing constructs beyond frequency (type, duration, intensity etc.); developing device-based assessments to improve measurement precision; and the inclusion of muscle-strengthening exercise into existing health surveillance systems.

## Introduction

Strong clinical and emerging epidemiological evidence shows that muscle-strengthening exercise (i.e. use of weight machines/body weight exercises) is independently associated with multiple health outcomes, including a reduced risk of all-cause mortality [1–3], incidence of diabetes [4, 5] and enhanced cardiometabolic [6, 7], musculoskeletal [8–10] and mental health [11, 12]. However, recent health surveillance data from multiple countries suggest only 10-30% adults meet the muscle-strengthening exercise guideline (≥ 2 sessions/week) [13–18]. Moreover, despite its numerous independent health benefits, in comparison to aerobic physical activity (e.g. walking, running or cycling), muscle-strengthening exercise has been largely overlooked in public health approaches for chronic disease prevention [14, 17, 19]. This current opinion paper:

i.Provides a narrative review of the emerging discipline of muscle-strengthening exercise epidemiology

ii.Argues the case for an increased emphasis on the role of muscle-strengthening exercise for chronic disease prevention

iii.Discusses key research gaps and strategies to advance this field

## Defining Muscle-strengthening Exercise

Muscle-strengthening exercise, sometimes referred to as strength/weight/resistance training or exercise, is a voluntary activity that includes the use of weight machines, exercise bands, hand-held weights, or own body weight (e.g. push-ups or sit-ups) [20]. When performed regularly, clinical exercise studies show that muscle-strengthening exercise increases skeletal muscle strength, power, endurance and mass [21]. This exercise-related behaviour is usually performed during a person’s leisure time, commonly within community (fitness centres/gyms) or home settings [22]. An individual may engage in muscle-strengthening exercise for numerous purposes, including for strength-related sports (e.g. weight/power lifting), aesthetic purposes (e.g. body-building/sculpting); physical therapy (e.g. rehabilitation from injury); conditioning for sports performance and for general fitness and health [23].

## Muscle-strengthening Exercise and Physical Activity Guidelines—a Recent Addition

Since the 1970s, physical activity recommendations for public health focused on promoting moderate-to-vigorous intensity aerobic physical activity (MVPA: e.g. walking, running or cycling) [24]. However, over the past decade, muscle-strengthening exercise has since been adopted. Muscle-strengthening exercise was initially included in the ‘2008 Physical Activity Guidelines for Americans’ [25], subsequently adopted in the World Health Organisation’s ‘2010 Global Recommendations on Physical Activity for Health’ [26] and is now included in many national public health recommendations [27–30]. The current global recommendations state that adults (18-64 years) should engage in:

i.At least 150 min of moderate-intensity aerobic physical activity, or at least 75 min of vigorous-intensity aerobic physical activity, or an equivalent combination of both a week

ii.Muscle-strengthening activities involving major muscle groups on 2 or more days a week [26]

Despite this inclusion, aerobic MVPA still remains the core focus of physical activity for chronic disease prevention [31–33]. The combination of muscle-strengthening exercise only being a recent addition into physical activity guidelines, and that it is still rarely assessed in health surveillance is likely to explain the current lack of research focus [16]. However, from a health promotion perspective, it might be possible that some may simply not ‘enjoy’ or find it difficult to engage in regular aerobic MVPA. For example, among those living in restrictive built environments lacking street connectivity, access to greenspace, and safe places to engage in common aerobic MVPA-related activities (e.g. walking, cycling and running) [34, 35]. Moreover, this exercise modality may be effective for those that are not able to perform aerobic MVPA due to co-morbidities, such as functional limitations or chronic obstructive pulmonary disease [36, 37]. Hence, there is a need to promote alternative forms of physical activity/exercise beyond aerobic MVPA [36, 37].

## Muscle-strengthening Exercise and Health

The addition of muscle-strengthening exercise into physical activity recommendations is due to the strong scientific evidence showing that this physical activity mode has multiple and unique health benefits. Since comprehensive reviews on muscle-strengthening exercise and health outcomes are available elsewhere [20, 21, 23, 38], we will only briefly discuss this evidence base.

### First, the Clinical

The health benefits of muscle-strengthening exercise from a clinical perspective are well established from over 30 years of research [38]. In brief, meta-analyses of short-duration clinical exercise studies show that muscle-strengthening exercise increases skeletal muscle mass/strength [23, 39, 40], bone mineral density [9, 41], the ability to perform activities of daily living [42], improves cardiometabolic health [6, 43] and reduces symptoms of depression/anxiety [11, 12]. In many of these meta-analyses, the benefits of muscle-strengthening exercise are independent of, or in some cases more effective than, aerobic MVPA [6, 11, 21, 39]. In addition, compared to aerobic MVPA, muscle-strengthening exercise has greater effects on emerging health conditions, such as preventing/treating sarcopenia [10] and maintaining physical function [44, 45]. This is particularly important when considering the current demographic trend of an ageing population [46], with declines in muscle mass/function projected to be amongst the key twenty-first-century public health challenges [10, 44, 45].

### Now, the Epidemiological

A limitation of findings from clinical exercise studies, even at the meta-analytical level, is the inclusion of small and homogeneous samples [6, 11]. From a public health perspective, it is necessary to establish how health benefits observed in controlled exercise studies translate to free-living community-dwelling adults. However, in comparison to the decades of epidemiological research on aerobic MVPA [38, 47, 48], similar research on muscle-strengthening exercise is limited. Nonetheless, recently epidemiological studies on the association between muscle-strengthening exercise and health have begun to emerge. A brief overview of the latest evidence now follows.

Saeidifard et al. conducted the first meta-analysis on the associations of muscle-strengthening exercise with mortality [1]. That analysis of 11 longitudinal studies (370,256 participants; mean follow-up = 8.85 years) showed that compared to no exercise, muscle-strengthening exercise was independently associated with 21% lower risk of all-cause mortality (after adjusting for aerobic MVPA, age, sex) [1]. Interestingly, a sub-analysis showed that compared to no muscle-strengthening exercise, 1-2 sessions/week was associated with reduced risk of mortality, whereas ≥ 3 sessions/week was not [49] suggesting that high doses (above the current guideline) of muscle-strengthening exercise may not necessarily be protective against morality. Since that meta-analysis, other longitudinal studies have shown that muscle-strengthening exercise is independently associated with reduced mortality risk [2, 3]. Prospective data from the US cohort studies have also identified that compared to those doing none, muscle-strengthening exercise is independently associated with a reduced incidence of diabetes [4, 5], cardiovascular disease [50], colon/kidney cancer [51], and gains in waist circumference [52].

### Run, Lift or Both?—Emerging Epidemiological Evidence for Combining Aerobic MVPA and Muscle-strengthening Exercise

In addition to these independent health benefits, our recent epidemiological studies suggest that, compared to engaging in either the muscle-strengthening exercise guideline (≥ 2 sessions/week) or the aerobic MVPA guideline alone (≥ 150 min/week), the combination of both (as is prescribed in the current guideline) may be most beneficial for the prevention and/or management of multiple prevalent chronic health conditions [14, 49, 53–58]. Our cross-sectional studies, amongst large samples (range: ~ 10,000 to ~ 1.7 million adults) across several countries (e.g. the USA, Germany and South Korea) have shown that compared to meeting the aerobic MVPA or muscle-strengthening exercise guideline alone, meeting both guidelines was associated with several important indicators of health. These include a reduced prevalence of cardiometabolic (hypertension, diabetes, cardiovascular disease) and general health conditions (arthritis, chronic obstructive pulmonary disease, asthma) [54, 58]; depression/depressive symptom severity [53, 56, 57]; obesity [49]; and prevalence of hyperglycaemia and dyslipidaemia [55]. Given the cross-sectional nature of these data, we urge caution in drawing strong causal inferences. Nonetheless, our findings are consistent with evidence from clinical studies demonstrating that, compared to engaging in either activity alone, combining aerobic MVPA and muscle-strengthening exercise has more favourable effects on cardiometabolic biomarkers [59–61], gains in lean muscle mass [62] and indicators of mental health [63].

## Assessment, Prevalence and Correlates of Muscle-strengthening Exercise in Health Surveillance

Whilst research on the assessment, prevalence and correlates of physical activity has historically focused on aerobic MVPA [31–33, 64–66], over the past decade, there has been some focus on the descriptive epidemiology of muscle-strengthening exercise [13, 14, 16]. We provide a brief overview of the common ways muscle-strengthening exercise is assessed in health surveillance, and the latest research on its prevalence and correlates.

### Assessment

In health surveillance, muscle-strengthening exercise is exclusively assessed by self-report, typically assessing its frequency only (sessions/week). In contrast to aerobic MVPA, there is currently no available validated device-based assessment method, such as accelerometry, to assess muscle-strengthening exercise in large population studies. Consequently, since self-reporting assessments of physical activity are prone to issues with social desirability and/or over reporting [67], muscle-strengthening exercise prevalence estimates obtained by self-report are likely to be overestimations [14]. Nonetheless, compared to aerobic MVPA, it is likely that individuals are able to more reliably recall engagement in muscle-strengthening exercise [68]. Yore et al. (2007) compared the reliability of survey items assessing both aerobic MVPA and muscle-strengthening exercise used in the US behavioural risk factor surveillance system survey, the largest and most consistently implemented survey assessing both exercise modalities [68]. That study showed that reliability estimates for muscle-strengthening exercise (Cohen’s kappa [*k*] = 0.85), exceed those for aerobic MVPA (*k* = 0.67) [68].

### Prevalence

The available studies on public health surveillance data (sample size range: ~ 9,000 to ~ 1.7 million adults) from several countries (e.g. the USA, Australia, Finland, the UK and Germany) suggest that between 10 and 30% of adults meet the muscle-strengthening exercise guideline (≥ 2 sessions/week) [13–18]. Moreover, our recent paper on trends of muscle-strengthening exercise amongst US adults suggests that at the population level muscle-strengthening exercise levels were stable between 2011 and 2017 (29.1 to 30.3%) [69].

**Fig. 1:**
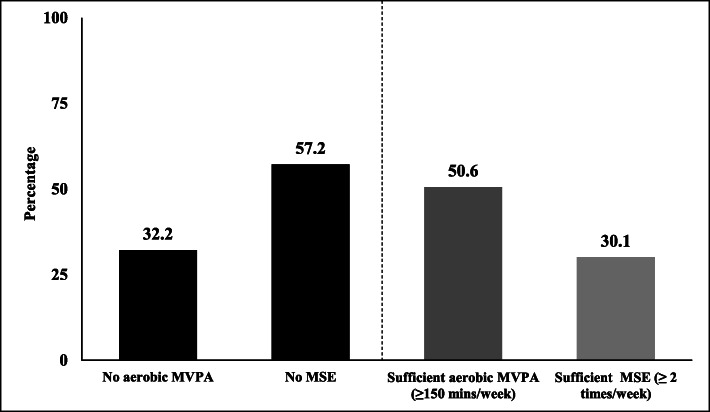
Percentages of adults (≥ 18 years; *n* = 1,677,108) reporting ‘No’ or ‘Sufficient’ moderate-to-vigorous intensity aerobic physical activity (MVPA: e.g. walking, running or cycling) and muscle-strengthening exercise (MSE; weight machines/body weight exercises)*. The asterisk indicates that data for this figure are drawn from pooling the 2011, 2013, 2015, and 2017 behavioural risk factor surveillance system surveys. Data available from Centers for Disease Control and Prevention Data and Documentation Repository: https://www.cdc.gov/brfss/data_documentation/index.htm

Compared to the proportions meeting the muscle-strengthening exercise guideline, the prevalence of those reporting sufficient aerobic MVPA guideline is considerably higher (~ 50%) [13, 15, 54]. Importantly, as shown in Fig. 1, our data amongst ~ 1.7 million US adults indicates that almost twofold greater proportions of US adults report no muscle-strengthening exercise (57.2%), compared to no aerobic exercise (32.2%) [49]. A potential explanation for these vastly differing prevalence levels is the fact that compared to certain types of aerobic physical activity/exercise that are common in daily living (e.g. walking for transport purposes/shopping), individuals have limited opportunity to engage in unintentional/incidental muscle-strengthening exercise. Based on this comparison, we argue that when paralleled to aerobic MVPA, equal (or possibly, greater) public health emphasis should be placed on the development of strategies and large-scale interventions to support the uptake/adherence of muscle-strengthening exercise at the population level [16, 54, 56, 57]. However, muscle-strengthening exercise has rarely been the focus of physical activity promotion for public health [19], and has even been referred to as the ‘forgotten’ [17] or ‘neglected’ guideline [70].

### Correlates

At present, most research on the correlates of muscle-strengthening exercise has focused on sociodemographic and lifestyle-related factors. Studies have consistently shown that older age, being female, having low education/income and being overweight/obese are inversely independently associated with not meeting the muscle-strengthening exercise guideline [13–18]. Moreover, our Australian data suggest that compared to those living in metropolitan settings, those living in rural and remote regions are less likely to meet the muscle-strengthening exercise guideline [14]. A systematic review by Rhodes et al. found based on the current limited literature, intrapersonal factors such as self-efficacy, affective judgements and self-regulation, and interpersonal factors including programme leadership and subjective norms may have a key role in muscle-strengthening exercise adherence [71].

## Muscle-strengthening Exercise Promotion—a Challenging Prospect

Despite being recommended by global/national public health agencies [26, 27, 29, 38], muscle-strengthening exercise has been a limited focus for public health approaches in chronic disease prevention [17, 19, 54]. This lack of focus is likely due to the fact that muscle-strengthening exercise is a complex behaviour with multiple and unique health promotion challenges [16]. For example, optimal muscle-strengthening exercise progression requires a basic understanding of specific terminology (e.g. sets, repetitions, large-muscle groups) [21], access to basic equipment (resistance bands/barbells) [20], self-efficacy to perform muscle-strengthening exercise-related activities (squats, lunges, push-ups) [71] and the likelihood of multiple entrenched negative social norms (e.g. fear of injury/excessive muscle gain/hyper-masculine settings) [37, 72–74].

To address these complicated factors, it is likely that concurrent, coordinated and multiple-level strategies are needed [16]. Some of these may include the following:

-Providing educational programmes and materials that offer basic information on muscle-strengthening exercise and its importance for health. Such educational strategies should focus on the fact that muscle-strengthening exercise does not necessarily require expensive equipment or access to specialised professionals. This approach would be particularly useful for older adults and those who are home-bound.

- Increasing the availability of equipment (barbells, resistance bands etc.) to encourage muscle-strengthening exercise in multiple settings (home, workplace etc.).
- Providing affordable/attractive spaces for muscle-strengthening exercise (community health clubs/centres, machines in open spaces).
- Enabling affordable public access to professionals who have skills in prescribing muscle-strengthening exercise (exercise physiologists/fitness instructors/strength coaches).
- Using behaviour-change science techniques to understand how different activities suit different sub-groups (e.g. older adults, culturally/linguistically diverse populations).
- Providing mass media campaigns endorsing muscle-strengthening exercise as important for health, and challenging its negative stereotypes.

## Moving Forward—Next Phases in Muscle-strengthening Exercise Epidemiology

Since research on the muscle-strengthening exercise epidemiology is still in its initial stages, there are multiple areas for future research. Some potential priority areas include the following:

### Standardising Assessment

Amongst studies of nationally representative samples, prevalence estimates for meeting the muscle-strengthening exercise guideline ranged from ~ 10% in Australia [14] to ~ 30% in the USA [16, 18]. Whilst this may be reflective of diverse muscle-strengthening exercise levels across countries, it is more likely that these differences are a consequence of the different surveillance instruments used across studies. Researchers should consider developing standardised muscle-strengthening exercise assessment items, as this would enhance the validity of cross-country comparisons and assist in accurately tracking/monitoring muscle-strengthening exercise levels.

### Beyond Frequency

At present muscle-strengthening exercise assessment items used in public health research predominantly only assess its frequency. Since clinical exercise studies demonstrate duration, intensity and type (single vs. multi-joint; body weight vs. use of weight machines etc.) of muscle-strengthening exercise may affect outcomes such as skeletal muscle strength/size/endurance [23], items that assess these muscle-strengthening exercise participation constructs will provide a more nuanced insight into this exercise modality and its associations with health.

### Device-based Assessments

As noted, a key limitation of assessment of muscle-strengthening exercise surveillance is that it is exclusively assessed by self-report. Whilst being of low cost/participant burden, self-report assessment of health behaviours is prone to issues with recall bias (e.g. social desirability and over/under reporting) [67]. Whilst currently unavailable, future studies should explore the use of wearable technologies/smart phone applications and their potential to assess muscle-strengthening exercise with greater precision.

### Beyond Sociodemographic Correlates

Congruent with the expansive research on the correlates of aerobic MVPA [31, 64], research should assess the potential for a wider range of possible influences, such as social (e.g. social norms/behavioural modelling) and physical environmental (e.g. access to facilities/equipment) factors. Moreover, future studies should examine the key barriers and facilitators amongst population sub-groups most at risk of low muscle-strengthening exercise engagement (e.g. older adults, females, those experiencing sociodemographic disadvantage).

### More Surveillance

Despite being globally recommended for a decade, muscle-strengthening exercise is still rarely assessed in physical activity surveillance [19]. As with common practice for aerobic MVPA [32, 33, 66], there is a need for surveillance systems to provide large-scale cross-country assessments of muscle-strengthening exercise. Such information is essential for the tracking and monitoring of this important health behaviour and establishing at risk population sub-groups for low-level engagement.

## Conclusion

This current opinion paper presents an overview of the emerging discipline of muscle-strengthening exercise epidemiology. The current scientific evidence indicates that the multiple and independent health benefits of muscle-strengthening exercise from a clinical perspective are strong, and rapidly emerging from an epidemiological standpoint. Importantly, epidemiological evidence suggests that amongst those doing none, small-to-moderate increases in muscle-strengthening exercise at the population level are likely to have considerable public health benefits. Yet, current conservative population estimates suggest that between 10-30% of adults report meeting the muscle-strengthening exercise guideline, a far lower proportion than those meeting the MVPA guideline (~ 50%). Success in large-scale interventions adherence/adherence of the muscle-strengthening exercise guideline at the population level will likely be contingent upon several multi-level and concurrent approaches. Future muscle-strengthening exercise epidemiology research should consider developing standardised muscle-strengthening exercise assessments in health surveillance (assessing constructs beyond frequency), examining a wider range of the potential correlates of muscle-strengthening exercise, and integrating assessments of muscle-strengthening exercise into existing health surveillance systems.

## Data Availability

Not applicable

## Abbreviations

MVPA: Moderate-to-vigorous intensity aerobic physical activity
*K*: Cohen’s kappa

## References

[1] Saeidifard F, Medina-Inojosa JR, West CP, Olson TP, Somers VK, Bonikowske AR, et al. The association of resistance training with mortality: a systematic review and meta-analysis. Eur J Prev Cardiol. 2019;2047487319850718. 10.1177/2047487319850718 31104484

[2] Stamatakis E, Lee IM, Bennie J, Freeston J, Hamer M, O’Donovan G (2018). Does strength-promoting exercise confer unique health benefits? A pooled analysis of data on 11 population cohorts with all-cause, cancer, and cardiovascular mortality endpoints. Am J Epidemiol.

[3] Tarasenko YN, Linder DF, Miller EA (2018). Muscle-strengthening and aerobic activities and mortality among 3+ year cancer survivors in the U.S. Cancer Cause Control.

[4] Grontved A, Pan A, Mekary RA, Stampfer M, Willett WC, Manson JE (2014). Muscle-strengthening and conditioning activities and risk of type 2 diabetes: a prospective study in two cohorts of US women. PLoS Med.

[5] Grontved A, Rimm EB, Willett WC, Andersen LB, Hu FB (2012). A prospective study of weight training and risk of type 2 diabetes mellitus in men. Arch Intern Med.

[6] Ashton RE, Tew GA, Aning JJ, Gilbert SE, Lewis L, Saxton JM. Effects of short-term, medium-term and long-term resistance exercise training on cardiometabolic health outcomes in adults: systematic review with meta-analysis. Br J Sports Med. 2018:bjsports-2017-098970. 10.1136/bjsports-2017-098970 29934430

[7] Lemes ÍR, Ferreira PH, Linares SN, Machado AF, Pastre CM, Netto J. Resistance training reduces systolic blood pressure in metabolic syndrome: a systematic review and meta-analysis of randomised controlled trials. Br J Sports Med. 2016:bjsports-2015-094715. 10.1136/bjsports-2015-094715 PMC5136729 26964146

[8] Schoenfeld BJ, Ogborn D, Krieger JW (2016). Effects of resistance training frequency on measures of muscle hypertrophy: a systematic review and meta-analysis. Sports Med.

[9] Martyn-St James M, Carroll S (2010). Effects of different impact exercise modalities on bone mineral density in premenopausal women: a meta-analysis. J Bone Miner Metab.

[10] Mcleod JC, Stokes T, Phillips SM. Resistance exercise training as a primary countermeasure to age-related chronic disease. Front Physiol. 2019;10(645). 10.3389/fphys.2019.00645 PMC6563593 31244666

[11] Gordon BR, McDowell CP, Hallgren M, Meyer JD, Lyons M, Herring MP (2018). Association of efficacy of resistance exercise training with depressive symptoms: meta-analysis and meta-regression analysis of randomized clinical trials. JAMA Psychiatry.

[12] Gordon BR, McDowell CP, Lyons M, Herring MP. The effects of resistance exercise training on anxiety: a meta-analysis and meta-regression analysis of randomized controlled trials. Sports Med. 2017:1–12. 10.1007/s40279-017-0769-0 28819746

[13] Bennie JA, Pedisic Z, Van Uffelen JG, Banting LK, Gale J, Vergeer I (2016). The descriptive epidemiology of total physical activity, muscle-strengthening exercises and sedentary behaviour among Australian adults – results from the National Nutrition and Physical Activity Survey. BMC Public Health.

[14] Bennie JA, Pedisic Z, van Uffelen JGZ, Charity MJ, Harvey JT, Banting LK (2016). Pumping iron in Australia: prevalence, trends and sociodemographic correlates of muscle strengthening activity participation from a national sample of 195,926 adults. PLoS ONE.

[15] Bennie JA, Pedisic Z, Suni JH, Tokola K, Husu P, Biddle SJ, et al. Self-reported health-enhancing physical activity recommendation adherence among 64,380 Finnish adults. Scand J Med Sci Sports. 2017. 10.1111/sms.12863 28230924

[16] Bennie JA, Lee D-C, Khan A, Wiesner GH, Bauman AE, Stamatakis E, et al. Muscle-strengthening exercise among 397,423 U.S. Adults: prevalence, correlates, and associations with health conditions. Am J Prev Med. 2018. 10.1016/j.amepre.2018.07.022 30458949

[17] Strain T, Fitzsimons C, Kelly P, Mutrie N (2016). The forgotten guidelines: cross-sectional analysis of participation in muscle strengthening and balance & co-ordination activities by adults and older adults in Scotland. BMC Public Health.

[18] Centers for Disease Control and Prevention (2013). Adult participation in aerobic and muscle-strengthening physical activities--United States, 2011. Centers for Disease Control and Prevention. MMWR Morb Mortal Wkly Rep.

[19] Milton K, Ramirez Varela A, Foster C, Strain T, Cavill N, Mutrie N (2018). A review of global surveillance on the muscle strengthening and balance elements of physical activity recommendations. J Frailty Sarcopenia Falls.

[20] American College of Sports Medicine (2009). American College of Sports Medicine position stand. Progression models in resistance training for healthy adults. Med Sci Sports Exerc.

[21] Garber CE, Blissmer B, Deschenes MR, Franklin BA, Lamonte MJ, Lee IM (2011). American College of Sports Medicine position stand. Quantity and quality of exercise for developing and maintaining cardiorespiratory, musculoskeletal, and neuromotor fitness in apparently healthy adults: guidance for prescribing exercise. Med Sci Sports Exerc.

[22] Loustalot F, Carlson SA, Kruger J, Buchner DM, Fulton JE (2013). Muscle-strengthening activities and participation among adults in the United States. Res Q Exerc Sport.

[23] Haff GG, Triplett NT (2015). Essentials of strength training and conditioning 4th edition: human kinetics.

[24] Oja P, Titze S (2011). Physical activity recommendations for public health: development and policy context. EPMA J.

[25] U.S Department of Health and Human Services. 2008 Physical activity guidelines for Americans. Washington; 2008.

[26] World Health Organization (2010). Global recommendations on physical activity for health.

[27] Australian Goverment Department of Health (2014). Australia’s physical activity & sedentary behaviour guidelines for adults (18-64 years).

[28] The UKK Institute for Health Promotion (2016). UKK Institute’s physical activity pie.

[29] UK Chief Medical Officer (CMO). UK chief medical officers’ physical activity guidelines. 2019 [cited 2019 13.09]; Available from: https://assets.publishing.service.gov.uk/government/uploads/system/uploads/attachment_data/file/829841/uk-chief-medical-officers-physical-activity-guidelines.pdf.

[30] Federal Ministry for Health. National recommendations for physical activity and physical activity promotion. 2016 [cited 2019 12.06]; Available from: https://www.sport.fau.de/files/2015/05/National-Recommendations-for-Physical-Activity-and-Physical-Activity-Promotion.pdf.

[31] Bauman AE, Reis RS, Sallis JF, Wells JC, Loos RJ, Martin BW (2012). Correlates of physical activity: why are some people physically active and others not?. Lancet (London, England).

[32] Guthold R, Stevens GA, Riley LM, Bull FC. Worldwide trends in insufficient physical activity from 2001 to 2016: a pooled analysis of 358 population-based surveys with 1·9 million participants. Lancet Glob Health. 2018. 10.1016/S2214-109X(18)30357-7 30193830

[33] Hallal PC, Andersen LB, Bull FC, Guthold R, Haskell W, Ekelund U (2012). Global physical activity levels: surveillance progress, pitfalls, and prospects. Lancet (London, England).

[34] McCormack GR, Shiell A (2011). In search of causality: a systematic review of the relationship between the built environment and physical activity among adults. Int J Behav Nutr Phys Act.

[35] Sallis JF, Cervero RB, Ascher W, Henderson KA, Kraft MK, Kerr J (2006). An ecological approach to creating active living communities. Annu Rev Public Health.

[36] Winett RA, Carpinelli RN (2001). Potential health-related benefits of resistance training. Prev Med.

[37] Phillips SM, Winett RA (2010). Uncomplicated resistance training and health-related outcomes: evidence for a public health mandate. Curr Sports Med Rep.

[38] U.S. Department of Health and Human Services. Physical activity guidelines for Americans, 2nd edition. Washington, DC; 2018.

[39] Schoenfeld BJ, Ogborn D, Krieger JW (2017). Dose-response relationship between weekly resistance training volume and increases in muscle mass: a systematic review and meta-analysis. J Sports Sci.

[40] Ralston GW, Kilgore L, Wyatt FB, Baker JS (2017). The effect of weekly set volume on strength gain: a meta-analysis. Sports Med (Auckland, NZ).

[41] Martyn-St James M, Carroll S (2009). A meta-analysis of impact exercise on postmenopausal bone loss: the case for mixed loading exercise programmes. Br J Sports Med.

[42] Mangione KK, Miller AH, Naughton IV (2010). Cochrane review: improving physical function and performance with progressive resistance strength training in older adults. Phys Ther.

[43] Strasser B, Siebert U, Schobersberger W (2010). Resistance training in the treatment of the metabolic syndrome: a systematic review and meta-analysis of the effect of resistance training on metabolic clustering in patients with abnormal glucose metabolism. Sports Med (Auckland, NZ).

[44] Prado CM, Purcell SA, Alish C, Pereira SL, Deutz NE, Heyland DK (2018). Implications of low muscle mass across the continuum of care: a narrative review. Ann Med.

[45] Rizzoli R, Reginster JY, Arnal JF, Bautmans I, Beaudart C, Bischoff-Ferrari H (2013). Quality of life in sarcopenia and frailty. Calcif Tissue Int.

[46] Jaul E, Barron J (2017). Age-related diseases and clinical and public health implications for the 85 years old and over population. Front Public Health.

[47] Lee IM, Shiroma EJ, Lobelo F, Puska P, Blair SN, Katzmarzyk PT. Effect of physical inactivity on major non-communicable diseases worldwide: an analysis of burden of disease and life expectancy. Lancet (London, England). 2012;380. 10.1016/S0140-6736(12)61031-9 PMC3645500 22818936

[48] U.S. Department of Health and Human Services. Physical activity and health: a report of the surgeon general. In: U.S. Department of Health and Human Services CfDCaP, National Center for Chronic Disease Prevention and Health Promotion, editor. Atlanta, GA; 1996.

[49] Bennie JA, De Cocker K, Pavey T, Stamatakis E, Biddle SJH, Ding D. Muscle strengthening, aerobic exercise, and obesity: a pooled analysis of 1.7 million US adults. Obesity (Silver Spring, Md). 2019. 10.1002/oby.22673 31709754

[50] Shiroma EJ, Cook NR, Manson JE, Moorthy MV, Buring JE, Rimm EB (2017). Strength training and the risk of type 2 diabetes and cardiovascular disease. Med Sci Sports Exerc.

[51] Mazzilli KM, Matthews CE, Salerno EA, Moore SC (2019). Weight training and risk of 10 common types of cancer. Med Sci Sports Exerc.

[52] Mekary RA, Grøntved A, Despres J-P, De Moura LP, Asgarzadeh M, Willett WC (2015). Weight training, aerobic physical activities, and long-term waist circumference change in men. Obesity..

[53] Bennie JA, De Cocker K, Biddle SJH, Teychenne MJ. Joint and dose-dependent associations between aerobic and muscle-strengthening activity with depression: a cross-sectional study of 1.48 million adults between 2011 and 2017. Depress Anxiety. 2019. 10.1002/da.22986 31876971

[54] Bennie JA, De Cocker K, Teychenne MJ, Brown WJ, Biddle SJH (2019). The epidemiology of aerobic physical activity and muscle-strengthening activity guideline adherence among 383,928 U.S. adults. Int J Behav Nutr Phys Act.

[55] Bennie JA, Ding D, Khan A, Stamatakis E, Biddle SJH, Kim J. Run, lift, or both? Associations between concurrent aerobic–muscle strengthening exercise with adverse cardiometabolic biomarkers among Korean adults. Eur J Prev Cardiol. 2018;2047487318817899. 10.1177/2047487318817899 30861691

[56] Bennie JA, Teychenne M, Tittlbach S. Muscle-strengthening exercise and depressive symptom severity among a nationally representative sample of 23,635 German adults. J Affect Disord. 2020. 10.1016/j.jad.2020.01.172 32056889

[57] Bennie JA, Teychenne MJ, De Cocker K, Biddle SJH. Associations between aerobic and muscle-strengthening exercise with depressive symptom severity among 17,839 U.S. adults. Prev Med. 2019. 10.1016/j.ypmed.2019.02.022 30786252

[58] Bennie JA, Lee D-C, Brellenthin AG, De Cocker K. Muscle-strengthening exercise and prevalent hypertension among 1.5 million adults: a little is better than none. J Hypertens. 2020; Publish Ahead of Print. 10.1097/HJH.0000000000002415 32102048

[59] Bird SR, Hawley JA (2017). Update on the effects of physical activity on insulin sensitivity in humans. BMJ Open Sport Exerc Med.

[60] Church TS, Blair SN, Cocreham S, Johannsen N, Johnson W, Kramer K (2010). Effects of aerobic and resistance training on hemoglobin A1c levels in patients with type 2 diabetes: a randomized controlled trial. Jama..

[61] Mann S, Beedie C, Jimenez A (2014). Differential effects of aerobic exercise, resistance training and combined exercise modalities on cholesterol and the lipid profile: review, synthesis and recommendations. Sports Med.

[62] Wadden TA, Vogt RA, Andersen RE, Bartlett SJ, Foster GD, Kuehnel RH (1997). Exercise in the treatment of obesity: effects of four interventions on body composition, resting energy expenditure, appetite, and mood. J Consult Clin Psychol.

[63] Sillanpaa E, Hakkinen K, Holviala J, Hakkinen A (2012). Combined strength and endurance training improves health-related quality of life in healthy middle-aged and older adults. Int J Sports Med.

[64] Trost SG, Owen N, Bauman AE, Sallis JF, Brown W (2002). Correlates of adults’ participation in physical activity: review and update. Med Sci Sports Exerc.

[65] Sallis J, Owen N (1999). Physical activity and behavioral medicine.

[66] Guthold R, Strong T, Chatterji K, Morabia S (2008). Worldwide variability in physical inactivity - a 51-country survey. Am J Prev Med.

[67] Dishman R, Heath G, Lee I (2012). Physical activity epidemiology (2nd edition).

[68] Yore MM, Ham SA, Ainsworth BE, Kruger J, Reis JP, Kohl HW (2007). Reliability and validity of the instrument used in BRFSS to assess physical activity. Med Sci Sports Exerc.

[69] Bennie JA, Kolbe-Alexander T, Seghers J, Biddle SJH, De Cocker K. Trends in muscle-strengthening exercise among nationally representative samples of U.S. adults between 2011 and 2017. J Phys Act Health. 2020; In press (accepted February 25th, 2020). 10.1123/jpah.2019-0472 32283540

[70] Bennie J, Smith J, Marvos Y, Biddle S, Kolbe-Alexander T (2019). The neglected guideline - resistance exercise and public health. J Sci Med Sport.

[71] Rhodes RE, Lubans DR, Karunamuni N, Kennedy S, Plotnikoff R (2017). Factors associated with participation in resistance training: a systematic review. Br J Sports Med.

[72] Dworkin SL (2001). “Holding back”: negotiating a glass ceiling on women’s muscular strength. Sociol Perspect.

[73] Howe HS, Welsh TN, Sabiston CM (2017). The association between gender role stereotypes, resistance training motivation, and participation. Psychol Sport Exerc.

[74] Lavallee ME, Balam T (2010). An overview of strength training injuries: acute and chronic. Curr Sports Med Rep.

